# The progress of percutaneous left atrial appendage occlusion: A bibliometric analysis from 1994 to 2022

**DOI:** 10.1097/MD.0000000000037742

**Published:** 2024-04-05

**Authors:** Huiqi Zhai, Liang Kang, Yihua Li, Xinjun Zhao, Qingmin Chu, Rong Li

**Affiliations:** a The First Clinical Medical College of Guangzhou University of Chinese Medicine, Guangzhou, China; b Department of Cardiovascular Disease, The First Affiliated Hospital of Guangzhou University of Chinese Medicine, Guangzhou, China.

**Keywords:** bibliometric analysis, left atrial appendage occlusion, oral anticoagulants, research hotspots

## Abstract

**Background:**

Atrial fibrillation is the most common cardiac arrhythmia, affecting 32 million individuals worldwide. Although atrial fibrillation has been studied for decades, a comprehensive analysis using bibliometrics has not been performed for atrial fibrillation-left atrial appendage occlusion (LAAO). Therefore, we analyzed the scientific outputs of global LAAO research and explored the current research status and hotpots from 1994 to 2022.

**Methods:**

We searched the Web of Science core collection for publications related to LAAO that were published between 1994 and 2022. We then performed bibliometric analysis and visualization using Microsoft Excel 2021, Bibliometric (https://bibliometric.com), VOSviewer (version 1.6.19), CiteSpace (version 6.2. R2), and the Bibliometrix 4.0.0 Package (https://www.bibliometrix.org) based on the R language were used to perform the bibliometric analysis, trend and emerging foci of LAAO in the past 29 years, including author, country, institution, journal distribution, article citations, and keywords. In total, we identified 1285 eligible publications in the field of LAAO, with an increasing trend in the annual number of publications.

**Results:**

The United States is the country with the most published articles in this field, while the United Kingdom is the country with the most cited literature. Mayo Clinic, from the United States, has the most publications in this area and Horst Sievert from Germany had the highest number of individual publications. The analysis of keywords showed that fibrillation, stroke, safety, oral anticoagulants, and watchman were the main hotpots and frontier directions of LAAO. Surgical treatment of nonvalvular atrial fibrillation, upgrading of related surgical instruments, and anticoagulation regimen after surgical treatment are the major research frontiers.

**Conclusion:**

We show that the research of percutaneous LAAO has been increasing rapidly over the last decade. Our aim was to overview past studies in the field of LAAO, to grasp the frame of LAAO research, and identify new perspectives for future research.

## 1. Introduction

Population aging brings to the attention of cardiologists increasingly complex patients with multiple comorbidities, such as atrial fibrillation, heart failure, and structural heart disease are increasingly becoming common cardiac conditions in the elderly population. Device therapy is becoming an important treatment option for these diseases. Therefore, combined percutaneous procedures are important in the field of interventional cardiology.^[1]^

Atrial fibrillation (AF) is the most common cardiac arrhythmia, affecting 32 million people worldwide. Its incidence increases with age, reaching approximately 6% in people >60.^[2]^ It is characterized by grossly disorganized atrial electrical activity and an increased thromboembolic risk. It is responsible for 15% to 20% of cerebrovascular accidents of ischemic origin. It has a high impact on the costs of the health system.^[3]^ Owing to its potentially serious complications and widespread prevalence, AF is a substantial health problem that causes significant economic burden in both developed and developing countries.

The treatment of AF is mainly to prevent complications such as cerebral infarction caused by the blood clots it produces, and anticoagulant medication is currently used. In nonvalve AF (NVAF), 90% of blood clots form at the level of the left appendage, a small, ear-shaped sac in the muscle wall of the left atrium.^[4,5]^ The 2 main classes of anticoagulants in common use include vitamin K antagonists and newer oral anticoagulants. Anticoagulant therapy is the gold standard in reducing the risk of stroke, but about 1 in 10 patients do not receive anticoagulant therapy due to contraindications: previous bleeding, anemia, chronic renal failure, and liver cirrhosis. The current guidelines recommend anticoagulant therapy in patients with CHA2DS2-VASc score ≥2. However, the risk of bleeding with oral anticoagulants has led to poor compliance in a large number of patients, such as in a study^[6]^ that showed that only 40% of 24,000 chronic kidney disease patients aged 65 years with AF received anticoagulation. Meanwhile, in a real-world study,^[7]^ up to 25% to 30% of patients stop OAC on long-term follow-up.

For such situation, the advent of percutaneous left atrial appendage occlusion (LAAO) offers a new way of treating patients with AF. In the 2016 ESC guideline and the 2019 ACC guideline, percutaneous LAAO is recommended for stroke prevention in patients with AF who are at high risk of stroke but have a long-term contraindication to anticoagulation (class IIb, level B). The 2022 Chinese expert consensus on AF also states that one-stop catheter ablation combined with percutaneous LAAO is a reasonable strategy for patients with indications (class IIa, level A). All these guidelines recommend percutaneous LAAO as an alternative to anticoagulation in patients with AF to varying degrees, providing more options for patients with AF.

For such cases, the advent of left auricular blockade offers a new approach to the treatment of patients with AF. In both the 2022 ESC guidelines^[8]^ as well as the 2019 ACC guidelines,^[9]^ LAAO is recommended for stroke prevention in patients with AF at high risk of stroke but with long-term contraindications to anticoagulation. The above guidelines propose to recommend LAAO as an alternative to anticoagulation for AF patients at high risk of stroke, providing more options for AF patients.

Over the past 3 decades, our understanding of AF has advanced greatly. However, these studies have not been measured systematically, with only a few bibliometric research focused on cardiovascular diseases.^[10,11]^ An overview of these literature was performed with the help of bibliometrics analysis software, we hope there were important values in our results, which could be positive for the development of AF research and we could provide some suggestions and inspirations to the researchers who work on AF research and treatment.

## 2. Methods and materials

### 2.1. Data source and search strategy

The literature related to percutaneous LAAO was retrieved from the web of science core collection (WOSCC) database. The retrieval work was completed within the day of July 6, 2023, to avoid inconsistent results due to database updates.

The searching parameters were: (LAAO); time span: 1994 to 2022; language type: English; literature type: Article and Review literature; index: sci-expanded, SSCI. After that, we derived a clustered network of 1285 articles. Bibliometric (https://bibliometric.com), VOSviewer (version 1.6.19), CiteSpace (version 6.2. R2), and the Bibliometrix 4.0.0 Package (https://www.bibliometrix.org) based on the R language were used to perform the bibliometric analysis, trend and emerging foci of LAAO in the past 29 years, including author, country, institution, journal distribution, article citations, and keywords. Data extraction and analysis management were performed by Zhai Huiqi and Kang Liang respectively to ensure the accuracy and reliability of the data.

### 2.2. Bibliometric analysis

Microsoft Excel was used to analyze the trend of annual publications, while VOSviewer and the online bibliometric analysis platform were used to construct visualization maps.

Co-authorship network of authors, countries, institutions, and co-occurrence of keywords was identified by CiteSpace 5.7.R1 (Leiden University, Leiden, the Netherlands) through co-occurrence analysis. In the graphs performed by this software, each point represented 1 element, including author, country, institution, and keyword, whose size was represented by the size of points. Furthermore, the connection between points represented the appearance or co-citation relationship, and the number of interconnects appears to increase the thickness of the collaboration which represents the strength of co-occurrence or co-citation.

In this bibliometric analysis, VOSviewer was used to build the institution, author, co-cited author, and co-cited journal network visualization maps to understand the general information in the LAAO field.^[12]^ In the co-citation maps, different points represent different elements (co-cited References/Journals/Authors), and the size of points is proportional to the number of citations of the publications.^[13]^ The lines between points indicate co-citation relationships. The different colored points and lines represent different clusters or years.^[14]^

Parameters of CiteSpace were set as described below: time slicing (1994–2022), year per slice, term source (all selected), selection criteria (top 50%) and pruning (pathfinder), and visualization (cluster view static, display merged network). The size of each point in the plot meant frequencies of occurrence or citation. The thickness and color of the rings at ineach points indicated the number and corresponding time period of each occurrence or citation.

In the bibliometric analysis, the top 10 countries, institutions, journals, and researchers in terms of highest citations, etc, are analyzed and summarized using tables. And information on related journals and researchers is also analyzed in detail.^[15–19]^

The journal impact factors were retrieved from *Journal Citation Reports* of 2023. Since the data and information were all secondary data that were exploited on the open database (WOSCC), no informed consent and ethical approval were required for this research.

## 3. Results

### 3.1. Annual analysis of publications

**Figure 1.**
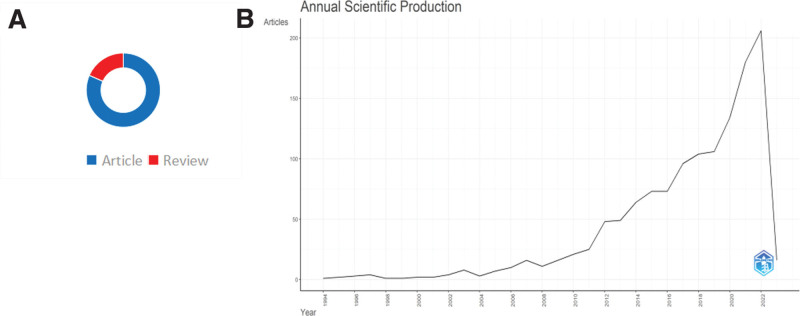
Type and annual distribution of publication results. (A) Document type. Blue represents articles, and red represents reviews. (B) Annual publications in the WoSCC database over the past 30 yr (excluding 2023). WoSCC = web of science core collection

A total of 1285 papers, including 1048 articles and 237 reviews, published between 1994 and 2022 on LAAO were identified in this study (Fig. 1A). Figure 1B presents the distribution of annual publications. Despite a downward trend at some points in time, the number of publications related to LAAO showed an overall upward trend, increasing from 134 in 2020 to 180 in 2021. In 2022, the highest number of publications was recorded, with 206 papers. The annual publication trend shows that researchers’ interest in LAAO has continued to increase over the past 20 years and continues to grow with positive momentum.

### 3.2. Co-authorship: countries, institutions, and authors

**Figure 2.**
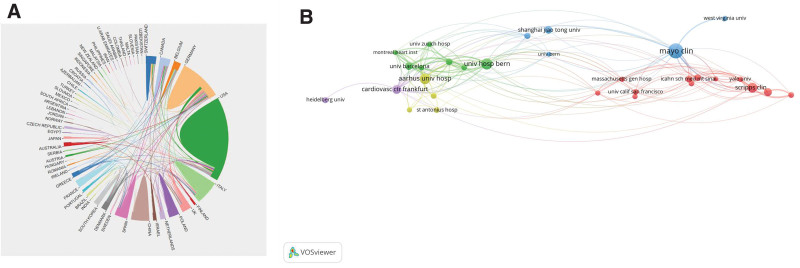
Cooperation between countries, institutions. (A) Co-authorship/cooperation between countries. (B) Co-authorship between institutions.

Nearly 45 countries around the world contributed to research on LAAO during the period 1994 to 2022. The United States (372 papers), Germany (172 papers), the People’s Republic of China (165 papers), Italy (72 papers), and Spain (52 papers) were the top 5 productive countries. The co-authorship between countries/institutions was shown (Fig. 2). Germany is the country that cooperates most closely with other countries. As shown in Table 1, Mayo Clinic (78 papers) was the institution that published the most productions, followed by Aarhus University Hospital (71 papers), University Hospital Bern (66 papers), ShangHai JiaoTong University (61 papers), and CardioVasculäres Centrum Frankfurt (52 papers). And then, it showed Horst Sievert (53 papers) from the CardioVascular Center Frankfurt ranked first, followed by David R Holmes Jr (42 papers), Xavier Freixa (38 papers), Bernhard Meier (34 papers), and Boris Schmidt (31 papers). Table 2 shows the most influential countries in terms of LAAO, with the United Kingdom being the most influential, followed by Greece, and then Israel, Canada, Germany, the United States, Switzerland, the Netherlands, India, and Australia.

**Table 1:** The top 10 active countries, institutions, and authors.

Rank	Country	Records	Institution	Records	Author	Records
1	the United States	372	Mayo Clinic	78	Horst Sievert	53
2	Germany	172	Aarhus university Hospital	71	David R Holmes Jr	42
3	China	165	University Hospital Bern	66	Xavier Freixa	38
4	Italy	72	Shanghai Jiao Tong University	61	Bernhard Meier	34
5	Spain	52	CardioVascular Center Frankfurt	52	Boris Schmidt	31
6	the United Kingdom	49	Texas Cardiac Arrhythmia Institute	52	Jens Erik Nielsen-Kudsk	30
7	Poland	46	Jagiellonian University	45	Andrea Natale	30
8	Switzerland	46	Schmidt	45	Apostolos Tzikas	29
9	Canada	43	Heidelberg University	44	Dhanunjaya Lakkireddy	29
10	France	33	University of Barcelona	39	Sergio Berti	28

**Table 2:** The top 10 countries with high academic influence.

Rank	Country	Average article citations
1	the United Kingdom	207.00
2	Greece	53.90
3	Israel	45.10
4	Canada	39.90
5	Germany	38.90
6	the United States	35.30
7	Switzerland	29.30
8	the Netherlands	24.80
9	India	24.50
10	Australia	23.30

### 3.3. Co-citation: references, journals, and authors

Co-cited references mean when both documents appear in the reference list of a third document, the 2 documents are said to be co-cited. Co-cited authors/journals were obtained from the co-cited references. The top 10 co-citations of Journals/Authors/References are in Tables 3–5. As the Table 3 demonstrates, the *Journal of the American College of Cardiology* is the journal with the highest number of cited articles (5440 records), followed by *Circulation* (3286 records), *New England Journal of Medicine* (1889 records), *European Heart Journal* (1821 records), and *Europace* (1563 records). Among the influences of the authors, Table 4 presents the top 10 cited authors on LAAO research. Among them, Vivek Y Reddy (2469 records) ranked first, followed by David R Holmes Jr (2355 records), Saibal Kar (1953 records), Maurice Buchbinder (1806 records), and Bernhard Meier (1645 records). Most of them are from the United States. Table 5 presents the top 10 cited references on LAAO research, 2016 ESC Guidelines for the management of AF developed in collaboration with EACTS, which is the most influential article with a record of 3386 citations.

**Table 3:** Top 10 journals with highest citations.

Rank	Journals	Citations	H-index	IF (2023)	Quartile in category
1	*Journal of the American College of Cardiology* (the United States)	5440	26	24.4	Q1
2	*Circulation* (the United States)	3286	13	37.8	Q1
3	*New England Journal of Medicine* (the United States)	1889	1	158.5	Q1
4	*European Heart Journal* (England)	1821	7	39.3	Q1
5	*Europace* (England)	1563	18	6.1	Q1
6	*Heart Rhythm* (the United States)	1420	17	5.5	Q2
7	*Lancet* (England)	1296	1	168.9	Q1
8	*Catheterization and Cardiovascular Interventions* (the United States)	1272	22	2.3	Q3
9	*EuroIntervention* (France)	1265	19	6.2	Q3
10	*Stroke* (the United States)	1228	5	8.4	Q1

**Table 4:** Top 10 authors with highest citations.

Rank	Authors	Citations	H-index	Location
1	Vivek Y Reddy	2469	17	the United States
2	Horst Sievert	2355	26	Germany
3	Shephal K Doshi	1953	11	the United States
4	David R Holmes Jr	1806	20	the United States
5	Saibal Kar	1645	15	the United States
6	Maurice Buchbinder	938	3	the United States
7	Bernhard Meier	812	18	Switzerland
8	Kurt Huber	771	5	Austria
9	Reda Ibrahim	744	16	Canada
10	Protect AF Investigators	738	2	the United States

**Table 5:** Top 10 references with highest citations.

Rank	References	Citations
1	2016 ESC Guidelines for the management of atrial fibrillation developed in collaboration with EACTS	3386
2	2020 ESC Guidelines for the diagnosis and management of atrial fibrillation developed in collaboration with the EACTS: the task force for the diagnosis and management of atrial fibrillation of the ESC developed with the special contribution of the EHRA of the ESC	3202
3	Percutaneous closure of the left atrial appendage versus warfarin therapy for prevention of stroke in patients with atrial fibrillation: a randomized noninferiority trial	1416
4	2012 focused update of the ESC Guidelines for the management of atrial fibrillation: an update of the 2010 ESC Guidelines for the management of atrial fibrillation. Developed with the special contribution of the European Heart Rhythm Association	1280
5	Prospective randomized evaluation of the Watchman Left Atrial Appendage Closure device in patients with atrial fibrillation versus long-term warfarin therapy: the PREVAIL trial	997
6	Safety of percutaneous left atrial appendage closure: results from the Watchman Left Atrial Appendage System for Embolic PROTECT AF clinical trial and the Continued Access Registry	587
7	5-yr outcomes after left atrial appendage closure: from the PREVAIL and PROTECT AF trials	501
8	Left atrial appendage closure with the Watchman device in patients with a contraindication for oral anticoagulation: the ASAP study (ASA Plavix Feasibility Study With Watchman Left Atrial Appendage Closure Technology)	485
9	Does the left atrial appendage morphology correlate with the risk of stroke in patients with atrial fibrillation? Results from a multicenter study	474
10	Percutaneous left atrial appendage closure for stroke prophylaxis in patients with atrial fibrillation: 2.3-yr Follow-up of the PROTECT AF (Watchman Left Atrial Appendage System for Embolic Protection in Patients with Atrial Fibrillation) trial	430

EACTS = European Association for Cardio-Thoracic Surgery, EHRA = European Heart Rhythm Association, ESC = European Association for Cardio-Thoracic Surgery, ESC = European Society of Cardiology, PROTECT AF = Protection in Patients with AF.

**Figure 3.**
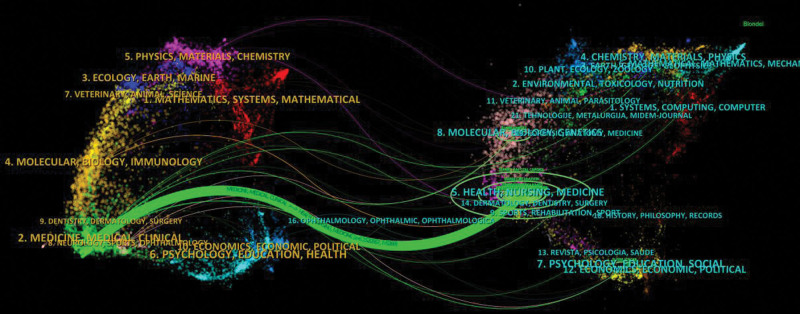
The dual-map overlay of articles citing on left atrial appendage occlusion research. (The left side is the citing journal, the right side is the cited journal, and the linepath represents the citation relationship.)

The dual-map overlay shows the main citation path. The published papers were mainly focused on journals in the field of physics, materials, chemistry, ecology, medicine, and clinical, whereas most of the cited articles were published in journals in the field of molecular, biology, genetics, health, nursing, and medicine (Fig. 3).

### 3.4. Co-occurrence analysis-keywords

**Figure 4.**
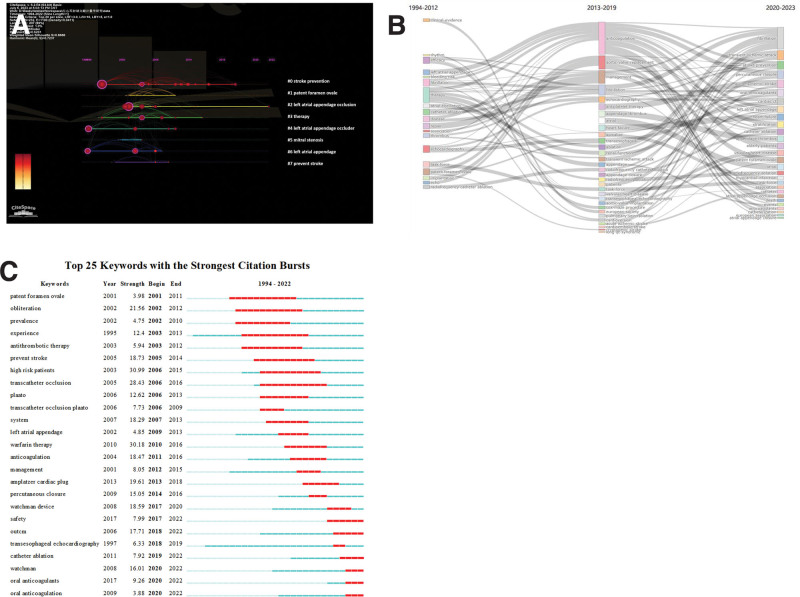
Visualization of keyword analysis. (A) Timeline distribution of cluster analysis of keywords. (B) Sankey diagram of the keywords evolution of LAAO research. (C) Representative burst keywords among top 25 references with the strongest citation bursts. LAAO = left atrial appendage occlusion.

**Figure 5.**
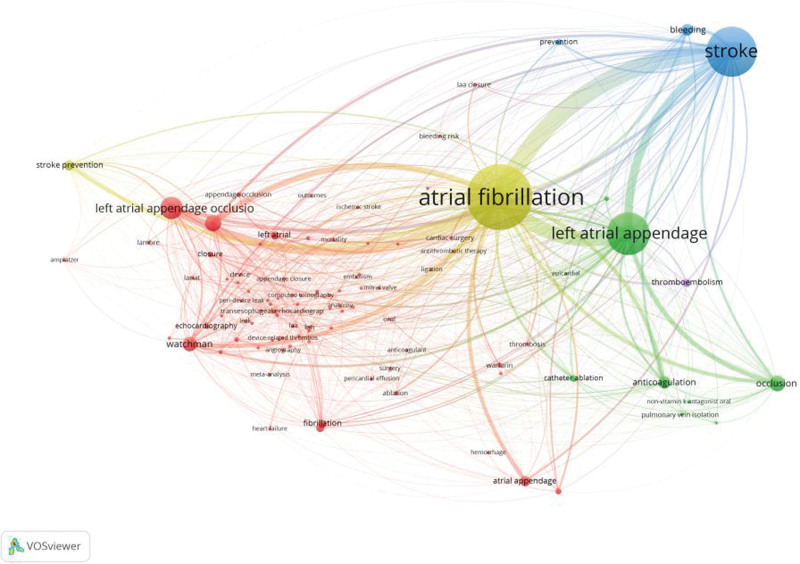
Keywords co-occurrence network knowledge of LAAO. LAAO = left atrial appendage occlusion.

The map of keywords on LAAO research was obtained by VOSviewer (Fig. 4). The modularity Q of the cluster was 0.6201, and the mean silhouette value was 0.8688. The timeline of clustering showed that “oral anticoagulation,” “catheter ablation,” “safety,” and “watchman device” were the most important areas of podocyte injury research, whereas “left atrial appendage occlusion” was an emerging hotspot in LAAO studies (Fig. 4A). We conducted a thematic evolution analysis on keywords, and found that the initial stages of research on LAAO were mainly focused on “therapy.” However, with the maturity of the research field, the main research hotspot of LAAO has gradually evolved toward “fibrillation,” “transient ischemic attack,” and “stroke prevention” among others. In the past 3 years, “anticoagulants” and “catheterization” among others, have gradually attracted the attention of scholars (Fig. 4B). In addition, we also found that “catheter ablation”, “oral anticoagulation” and “watchman” were the latest keywords that emerged in the last 3 years (Fig. 4C). And we also made a keyword co-occurrence network knowledge (Fig. 5). We can tell from the images that there is the closest relationship and highest density between LAAO and stroke and AF.

As shown in Table 6, the top 10 keywords used more than 1200 times were as follows: fibrillation (604 records), stroke (423 records), closure (377 records), occlusion (369 records), warfarin (341 records), risk (259 records), prevention (216 records), device (201 records), stroke prevention (200 records), and anticoagulation (168 records).

**Table 6:** Top 20 keywords in terms of records in LAAO research.

Rank	Keywords	Records	Rank	Keywords	Records
1	fibrillation	604	11	therapy	166
2	stroke	423	12	outcomes	140
3	closure	377	13	warfarin therapy	125
4	occlusion	369	14	high-risk patients	117
5	warfarin	341	15	management	114
6	risk	259	16	safety	112
7	prevention	216	17	watchman device	112
8	device	201	18	transcatheter occlusion	92
9	stroke prevention	200	19	watchman	86
10	anticoagulation	168	20	experience	85

LAAO = left atrial appendage occlusion.

## 4. Discussion

In this study, we conducted a bibliometric analysis of publications related to LAAO over the past 28 years so that we could identify key hotspots and trends in LAAO research. We have observed a gradual increase in publications related to LAAO since 2011. We observed a rapid increase in LAAO-related research, especially after 2019, indicating that this field will still remain a hot topic of interest for researchers in the coming years. In our study, we show that the United States is the country with the most published papers in this field, while the United Kingdom is the country with the most cited literature. With regards to international cooperation, Germany, the United States, and Italy were found to collaborate closely with other countries, while China and France collaborate to a lesser extent; this may be the reason for their low ranking in the number of citations. Perhaps cooperation between China and France should be promoted, so that higher-quality publications can be disseminated. Thankfully, there was a developing country called China in the top 10 countries, which represented that China had made great contributions to LAAO research in the past 10 years, and is becoming more and more powerful in this field.

Analysis of the top 10 most popular journals shows 50% were in the United States. Mayo Clinic, in the United States, has the most publications in this area, indicating that this institution publishes higher quality articles and could be considered for further collaboration and learning. For authors, Vivek Y Reddyy from New York, United States contributed the highest total citations, while Horst Sievert from Germany had the highest number of individual publications and all made significant contributions to the progression of LAAO research. Moreover, it is evident from the network that these teams maintain close collaboration with each other; this is why they can produce high-quality publications.

The most frequent co-cited reference named 2016 ESC Guidelines for the management of AF developed in collaboration with EACTS. The 2016 ESC systematically elucidated the diagnosis, assessment, and management of AF, among other things. The 2016 ESC systematically explains the diagnosis, assessment, and management of AF, further expands the indications for catheter ablation, and describes the value of LAAO in the treatment of AF. More importantly, the guideline puts forward new ideas on anticoagulation for patients with AF in combination with other diseases, and reinforces the significance of device therapy in the treatment of AF.

### 4.1. Current research hotpots

Based on the keywords co-occurrence analysis, we were able to find fibrillation, stroke, and warfarin are closely related to LAAO. LAAO is aimed at patients with AF who have a CHA2DS2-VASc score ≥2. The left atrial appendage (LAA) is considered to be a pocketed atrial appendage with abundant internal comb muscle, and when AF occurs, blood flow from the atria into the LAA is slowed down or stagnant, so it is prone to thrombus formation, which further triggers the development of cardiac thrombus.^[20]^

Cardiogenic embolism is closely related to stroke. Cardioembolic stroke secondary to AF accounts for 15% of all ischemic strokes.^[21]^ Therefore, in order to reduce the risk of stroke in patients with AF, reducing their thrombus presence is an important approach. The LAA is a finger-like or stump-like extension of the left atrial with lobes that may harbor up to 90% of thrombi that occur in patients with AF.^[4]^ In contrast to the smooth-walled left atrial, the LAA contains pectinate muscles that form a complex network of muscular ridges. Therefore, the implementation of occlusion therapy for the LAA can prevent the thrombus in the LAA from moving with the blood flow in the aorta and obstructing the blood vessel, which can cause stroke and other diseases. Therefore, LAAO has important clinical value in reducing the risk of stroke and improving the prognosis of patients with AF. Therefore, LAAO can prevent the thrombus in the LAA from moving with the blood flow in the aorta and blocking the blood vessel, which can cause stroke and other diseases.^[22]^ Therefore, LAAO has important clinical value in reducing the risk of stroke and improving the prognosis of patients with AF.

Top 25 keywords with the strongest citation bursts (Fig. 4C) show that oral anticoagulants and safety have become keywords that appear more and more frequently in the last 5 years. It suggests that the safety of oral anticoagulants and therapy in the treatment and management of AF is an issue that deserves constant attention and will likely remain a hot topic for researchers for some time to come.

Clinical research has shown that in patients with nonvalvular AF both novel oral anticoagulants and LAAO are safer than warfarin anticoagulation and not inferior to warfarin. A meta-analysis of randomized clinical trials of new oral anticoagulants versus warfarin showed a favorable risk-benefit profile, with significant reductions in stroke, intracranial hemorrhage, and mortality, and relative reductions in major hemorrhage of >66% or greater, providing a more reliable safety profile.^[23]^ A randomized clinical trial study of LAAO versus oral warfarin anticoagulation^[24]^ showed that LAAO was noninferior to warfarin for ischemic stroke prevention or SE >7 days’ postprocedure. Procedural safety has significantly improved. This trial provides additional data that LAAO is a reasonable alternative to warfarin therapy for stroke prevention in patients with nonvalve AF who do not have an absolute contraindication to short-term warfarin therapy.

### 4.2. Future frontiers

Based on our review of the timeline of research topics for LAAO before 2022, we forecast that 3 research topics will play significant roles in the future as follows.

#### 4.2.1. Surgical treatment of nonvalvular AF.

For the treatment of nonvalvular AF, the place of surgical treatment is becoming increasingly important. One-stop strategy for treatment of AF is being developed and implemented. “One-stop strategy” refers to the completion of multiple surgical procedures in a single procedure. Commonly, there is a one-stop strategy of LAAO and catheter-based AF ablation, a combination of LAAO and atrial septal defect/patent foramen ovale occlusion, and a combination of transcatheter aortic valve replacement and LAAO. A clinical trial from China^[25]^ showed that LAAO + catheter-based AF ablation on patients with AF can achieve rhythmic control and stroke prevention. The potential beneficiaries of this treatment regimen identified in the study are 60 to 70 years old age; CHA2DS2-VASc scores: male ≥2; female ≥3; left atrium diameter ≤48 mm; duration of AF  ≤3 years. Of course, with the accumulation of evidence, the characteristics of the indicated population will tend to be much clear and specific. For patients with AF with combined atrial septal defect or patent foramen ovale failure, one-stop occlusion is safe and effective. It has clinical significance in the prevention of stroke and deserves to be further promoted.^[26]^ And of course there are researchers who had operated (transcatheter valve replacement and LAAO) on patients with severe aortic stenosis combined with AF, and the results suggest that it is safe and feasible.^[27]^ These attempts provide new ideas and methods for clinical practitioners to learn and learn from in treating AF combined with other diseases.

#### 4.2.2. Upgrading of related surgical instruments.

The anatomical morphology of the LAA does not show homogeneity; common ones are chicken wing LAA, windsock LAA, cauliflower LAA, and cactus LAA, among others. There is also diversity in the opening pattern of the LAA, such as oval, foot-like, triangular, water drop-like, round, and so on.^[28]^ Because of the variety and irregularity of the anatomical morphology of the LAA and the shape of the opening, the current disc-shaped occluder is sometimes not able to cover the opening completely, so that in some patients blood flow still passes through the opening of the LAA, resulting in shunt phenomenon.^[29]^ Incomplete blockade (shunt >5 mm) can still lead to cardiogenic thrombosis, and oral anticoagulation will still be a daily task for these patients.^[30]^ To address these issues, some scholars have proposed Three/3 Dimensions modeling of the LAA opening and then Three/3 Dimensions printing to guide LAAO,^[31]^ while some scholars^[32]^ have proposed multi-angle and multi-dimensional occlusion guidance to achieve the best fit of the occluder disk to the LAA opening. However, we believe that, in addition to the above methods, upgrading the blocking device, customizing the blocker according to the anatomical structure of the patient’s LAA and the opening, or adopting an umbrella-type structure for the external disk to cover as wide a range of LAA openings as possible may be a direction or trend for future research.

#### 4.2.3. Anticoagulation regimen after surgical treatment.

Anticoagulation is the foundation and core of drug therapy for patients with AF. For patients with AF who undergo LAAO, such as those blocked with watchman, 45 days of postoperative anticoagulation is required, followed by a review of transesophageal echocardiography to rule out thrombus before switching to dual antiplatelet therapy for 6 months, and finally, maintenance aspirin for long-term treatment.^[33]^ However, a trial^[34]^ from the United States in 2022 showed that a dual antiplatelet therapy discharge regimen may be an acceptable alternative to the use of anticoagulation and aspirin. This trial also demonstrated an association between increased risk of adverse events, particularly bleeding events, associated with adding aspirin to anticoagulation upon discharge after LAAO device implantation, and questioned the protocol of warfarin in combination with aspirin. In view of the above research content, we found that there is still no standardized management protocol after LAAO, leading to the fact that in many cases the patient’s management protocol depends on the physician’s experience. Therefore, we predict that determining a rational management plan after LAAO will be a hot and focused issue in clinical research in the coming years.

### 4.3. Strengths and limitations

This was a bibliometric study in the research field of percutaneous LAAO. The visualization analysis provided insights for researchers to understand the research hotspots and development trends of LAAO.

However, this study also has some limitations. First, we only retrieved research articles from WOSCC and excluded conference articles, etc, so the comprehensive of the paper was limited. Second, since the same abbreviations of some authors’ names in articles, and bibliometrics software could not distinguish the contributions of author with the same name, loss of accuracy may still be inevitable despite we tried hard to correct them. Third, although we sorted out the main research contributions of the main literature, the analysis was still incomplete, and researchers are required to read the literature further to find more meaningful research directions.

## 5. Conclusion

In our research, our analysis identified specific countries, institutions, authors, and journals, that made significant contributions to this field of research during the study period. We show that the research of percutaneous LAAO has been increasing rapidly over the last decade. Our aim was to overview past studies in the field of LAAO, to grasp the frame of LAAO research, and identify new perspectives for future research.

## Acknowledgments

The authors appreciate the help of Yihua Li, Liang Kang, Xinjun Zhao, and Qingmin Chu in the whole process of the paper. And thanks to Professor Rong Li for the project administration.

## Author contributions

**Conceptualization:** Huiqi Zhai, Liang Kang, Rong Li.

**Data curation:** Huiqi Zhai, Liang Kang, Yihua Li.

**Formal analysis:** Huiqi Zhai, Xinjun Zhao, Qingmin Chu.

**Methodology:** Huiqi Zhai, Liang Kang, Qingmin Chu.

**Project administration:** Huiqi Zhai, Liang Kang, Xinjun Zhao.

**Validation:** Huiqi Zhai, Liang Kang.

**Visualization:** Huiqi Zhai, Xinjun Zhao, Rong Li.

**Writing—original draft:** Huiqi Zhai, Yihua Li.

**Resources:** Liang Kang, Yihua Li, Rong Li.

**Software:** Liang Kang, Xinjun Zhao.

**Writing—review & editing:** Liang Kang, Qingmin Chu, Rong Li.

**Supervision:** Yihua Li.

**Investigation:** Rong Li.
